# Animal models of membranous nephropathy: more choices and higher similarity

**DOI:** 10.3389/fimmu.2024.1412826

**Published:** 2024-10-21

**Authors:** Ying Pan, Si Chen, Lin Wu, Changying Xing, Huijuan Mao, Hongwei Liang, Yanggang Yuan

**Affiliations:** ^1^ Department of Nephrology, the First Affiliated Hospital of Nanjing Medical University, Nanjing Medical University, Nanjing, China; ^2^ School of Life Science and Technology, China Pharmaceutical University, Nanjing, China

**Keywords:** membranous nephropathy, end stage kidney disease, animal model, advantages, disadvantages

## Abstract

Membranous nephropathy (MN) is an antibody-mediated autoimmune glomerular disease in which PLA2R1 is the main autoantibody. It has become the most common cause of adult nephrotic syndrome, and about one-third of patients can progress to end-stage kidney disease, but its pathogenesis is still unclear. Animal models can be used as suitable tools to study the pathogenesis and treatment of MN. The previous Heymann nephritis rat model and C-BSA animal model are widely used to study the pathogenesis of MN. However, the lack of target antigen expression in podocytes of model animals (especially rodents) restricts the application. In recent years, researchers constructed animal models of antigen-specific MN, such as THSD7A, PLA2R1, which more truly simulate the pathogenesis and pathological features of MN and provide more choices for the follow-up researchers. When selecting these MN models, we need to consider many aspects, including cost, difficulty of model preparation, labor force, and whether the final model can answer the research questions. This review is to comprehensively evaluate the mechanism, advantages and disadvantages and feasibility of existing animal models, and provide new reference for the pathogenesis and treatment of MN.

## 1 Introduction

**Figure 1:**
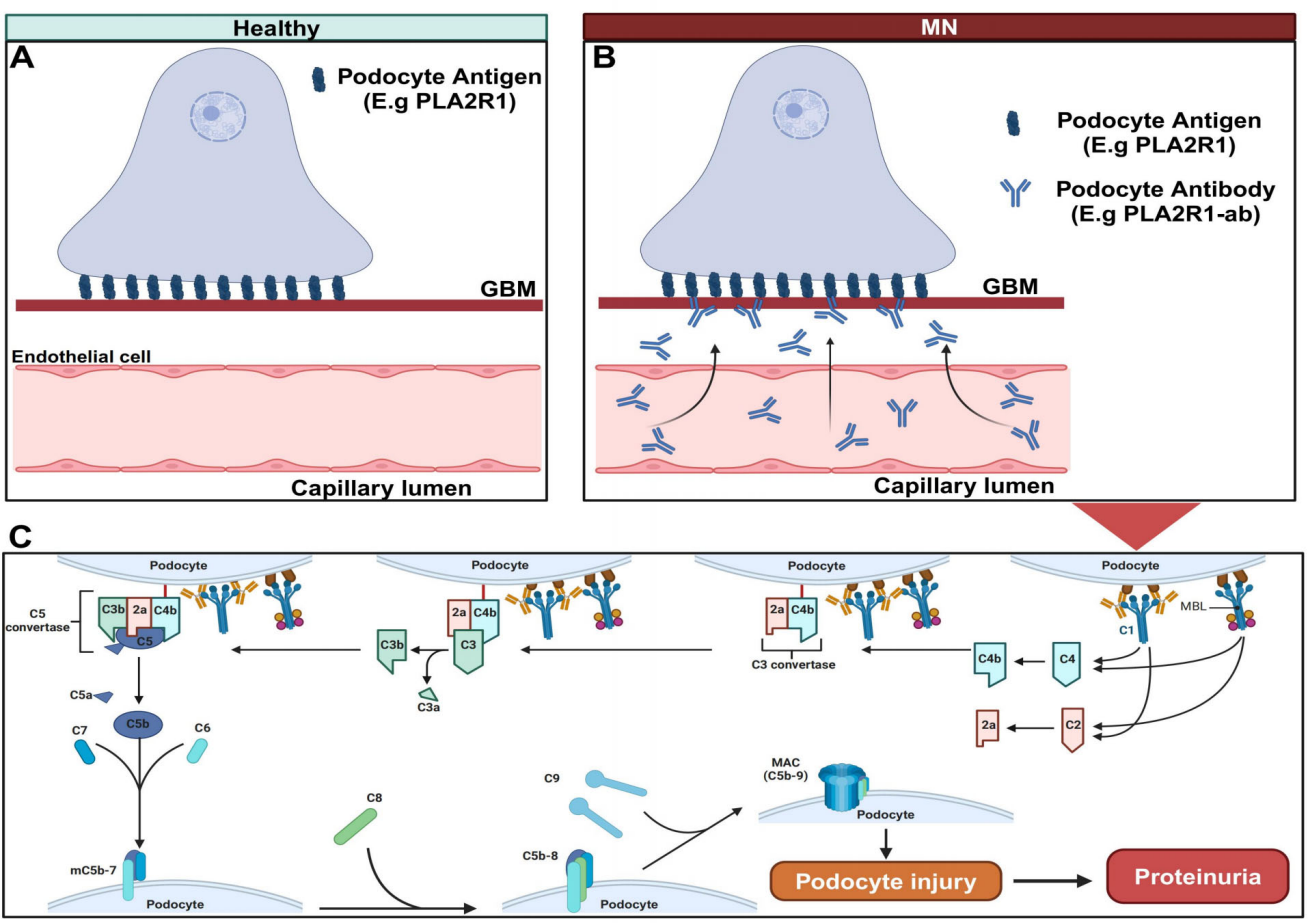
Pathogenesis of primary membranous nephropathy. **(A)** Normally, M-type phospholipase A2 receptor 1 (PLA2R1), Neural epidermal growth factor-like 1 protein (NELL-1) and thrombospondin type-1 domain-containing 7A (THSD7A) are expressed on glomerular podocytes. **(B)** Under pathological conditions, circulating antibodies combine with antigens on the surface of podocytes to form antigen-antibody complexes, which are deposited on the subepithelium and basement membrane to activate the complement pathway. **(C)** The classical complement pathway and lectin pathway are activated, eventually C5b-9 membrane attack complexes (MAC) is formed, which mediates podocyte damage and leads to a large number of proteinuria. Created with BioRender.com.

In recent years, the prevalence of renal disease, especially chronic renal disease, has increased year by year. Renal disease has become a serious public health problem, and its correlation with serious cardiovascular events has been widely recognized (1–3). The prevalence of chronic kidney disease in the population is 14.3%, and the prevalence in high-risk population is 36.1% (4). Nowadays, the incidence of membranous nephropathy (MN) has risen dramatically with a tendency to be younger (5, 6), which has become the most common cause of nephrotic syndrome in adults. Its pathological features are diffuse thickening of the glomerular basement membrane, granular deposition of IgG and complement C3 along the glomerular capillaries, and dense deposition of electrons formed by spikes in visceral epithelial cells of the glomerular basement membrane, which is mainly manifested as nephrotic syndrome or occult proteinuria in the clinic (7). Its pathogenesis has not been fully clarified. Most scholars believe that it is due to the deposition of immune complex caused by various reasons, which then activates complement and produces C5b-9 membrane attack complexes (MAC). (**Figure 1**) According to the etiology, it can be divided into primary membranous nephropathy (PMN) and secondary membranous nephropathy (SMN). The former is unknown, that is, the latter may be secondary to infection (hepatitis B and hepatitis C virus) (8, 9), systemic diseases (such as lupus erythematosus) (10), drug therapy (such as gold and penicillamine, etc.) (11) and malignant tumors (12).

However, the pathogenesis of MN is not fully understood, and there is also a lack of recognized treatments. Therefore, it is very important to understand the pathogenesis of MN, explore appropriate treatment methods and establish effective animal models of MN to study this disease. The animal models of MN in recent years were reviewed in detail below (**Table 1**).

**Table 1:** Comparison of different MN models.

Model	Animal		Successfully constructed animal strains	Duration	Dense deposits	Antibody type	Complement pathway	Antibody source	Target antigen	Correlation with human MN	Advantages	Disadvantages	References
Active Heymann nephritis (AHN)	Rat	Active (AHN)	SD; Lewis; Fisher et al.	8-10 weeks	Detected	Unknown	Classical pathway	Autologous	Megalin (gp330)、RAP	The antigen was expressed in human podocyte and proximal tubule, but no related antibody was detected.	First model to be developed, stable lesion, serious glomerular damage and obvious proteinuria, long course of disease is beneficial to long-term observation.	Time consuming	(13)
Passive Heymann nephritis (PHN)	Rat	Passive (PHN)	SD; Wistar et al.	7-10 days	Detected	Unknown	Classical pathway and Alternative Pathway	Allogenic	Fx1A	no related antibody was detected	Easy to model, less antibody consumption, less painful stimulation to animals, stable lesion, nearly identical pathology, short time frame.	Depending on exogenous antibodies, the disease is easy to alleviate, and long-term research requires repeated injection of antibodies, which is different from the pathogenesis of human membranous nephropathy.	(21, 22)
Anti-DPP IV model	Rat		SD; Lewis et al.	2-5 days	Detected	Rabbit-derived antibody	Nonactivated	Allogenic	DDP IV (gp108)	Expressed in human podocytes, while no related antibody was detected	Develop rapidly	Short maintenance time of proteinuria and IgG deposition, not as practical as HN model	(24)
C-BSA model	Mice,		ICR;BALB/c et al. (C57BL/6 is insensitive)	4-6 weeks	Detected	mIgG1>mIgG2	Unknown	Autologous	C-BSA	Mostly exists in children with MN, especially in infants under 5 years old.	Rich in species, easy to obtain, wide application, disease development of the mouse model is similar to that of MN patients, which depends on Th2 cells.	Antigen dose–response relations and strain differences, no definite evidence to support the view that antigen are related to human membranous nephropathy.	(32, 33)
	Rat		SD; Wistar et al.	8 weeks	Detected	Unknown	Unknown	Autologous	/				(29–31)
	Rabbit, dog, cat et al.			5-17 weeks	Detected	/	/	Autologous	/				(26–28)
Anti-α3NC1 model	mice		DBA/1; FcγR-/-; FcγRIII-/- et al.	6-18 weeks	Detected	mIgG1>mIgG2, mIgG3	Alternative Pathway	Autologous	α3NC1	As the main autoantigen of human anti-basement membrane glomerulonephritis, its relationship with MN has not been confirmed	Develop rapidly, stable lesion.	Less applications, low recognition, different strains have different sensitivities.	(37)
APN model	mice		C57BL/6N; 129S2/Sv; BALB/c; DBA/2 et al.	12-22 days	Detected	Goat/Rabbit-derived antibody	May be a complement-independent mechanism	Allogenic	Annexin-3	significant expression of Annexin-3 in the renal tissue of model mice and MN patients	Develop rapidly	Less applications, low recognition, maybe a complement-independent mechanism, mice need to be killed within 14 days, not conducive to long-term observation,	(41)
APA model	mice		BALB/c	0-16 days	Unknown	mIgG1	Unknown	Autologous	APA	Not found	Develop rapidly	Depending on passive antibody administration, complement activation was rare, and the above antigens were not present in human PMN, so they were significantly different from human PMN	(45)
THSD7A-associated model	mice		BALB/c	3-70 days	Detected	human-derived antibody	Lectin pathway	Allogenic	THSD7A	Very similar to human MN in proteinuria and histopathological changes	More similar to the pathogenesis of human PMN, long course of disease is beneficial to long-term observation	Strain differences; The detection rate of antigen is low, which may not represent the pathogenesis and all pathological changes of PMN.	(52)
	mice		BALB/c	0-14 days	Detected	Rabbit-derived antibody	Lectin pathway	Allogenic	THSD7A	Very similar to human MN in proteinuria and histopathological changes	Significant proteinuria was induced without complement activation.	The complement activation pathway is different from human PMN.	(53)
PLA2R1-associated mice model	mice	mPLA2R1	Transgenic mice	7 days	Detected	Rabbit-derived antibody	Unknown	Allogenic	mPLA2R1	Murine antigen, similar to human MN expression	First animal model expressing PLA2R1	Passive administration of exogenous antibodies, cannot produce spontaneously and combine with human antibodies. cannot explain the mechanism of autoantibody production in human PMN. Short course of disease is not conducive to long-term observation.	(62)
	mice	hPLA2R1	Transgenic mice	4-6 weeks	Detected	mIgG1>mIgG2, mIgG3	not classical pathway	Autologous	hPLA2R1	Expression of human MN antigen	Same antigen, produce spontaneously, ideal model to clarify the pathogenesis and antibody pathogenicity of human PMN.	Difficult to obtain; severe nephrotic syndrome appeared within 3-4 weeks after the appearance of antibody, cannot meet the needs of long-term observation.	(65)
	mice	chPLA2R1	Transgenic mice	12 weeks	Detected	mIgG1>mIgG2, mIgG3	All three pathways are involved	Autologous	chPLA2R1	Including three highly immunogenic human domains	Antigen specificity, immune-mediated character, similar complement activation pathway, stable lesion, the closest animal model to human MN so far.	Difficult to obtain, short research time	(71)
Anti-NEP model	Rabbit		/	/	Unknown	human-derived antibody	Unknown	Allogenic	NEP	NEP is the first human MN antigen discovered	Antigens are expressed in human podocytes	Less research	(75)
Anti-ACE associated minipigs model	minipig, Rabbit		minipig	/	Detected	Goat-derived antibody	Unknown	Allogenic	ACE	no related antibody was detected	Enrich the types of research	Less research	(76)
Anti-PLA2R1 associated minipigs model	minipig		minipig	1-2 weeks	Detected	human-derived antibody	Unknown	Allogenic	PLA2R1	Expresses the same antigen as human MN	Show the histological characteristics of early chronic nephropathy, easier to obtain than PLA2R1 mouse model, express the same antigen as human MN	In developmental stage, small sample size, low level of proteinuria	(77)

## 2 Rat model

### 2.1 The Heymann nephritis model

HN model is the most classic and widely used MN animal model at present. According to the antigen source, it can be divided into active Heymann nephritis (AHN) and passive Heymann nephritis (PHN). In 1959, HEYMANN et al. (13) injected allogenic or heterologous rat renal cortex homogenate and Freund’s complete adjuvant (which can be used as a non-specific immune system activator) into the abdominal cavity of rats. After 3-4 weeks, particle deposition of rat IgG in the glomerular capillary wall and subepithelial electronic dense deposits was observed, and after 8-10 weeks, 30-80% of rats showed significant proteinuria, which is the AHN model. The model has been successfully constructed in SD, Lewis, Fisher and other strains of rats. In the PHN model, brush border antigen (FxlA) of rats’ proximal renal tubular epithelial cells was injected into heterogeneous animals, such as rabbits. Then serum antibodies produced by immunized animals were extracted and injected into the tail veins of rats at one time. After injecting anti-Fx1A antibody for 3-5 days, the deposition of IgG, C3 and C5b-9 in the subepithelial tissue can be detected. After 7-10 days, persistent proteinuria appeared in rats.

The main pathogenic antigens of HN have been identified as megalin (also known as gp330) and receptor-associated protein (RAP) (14), which are members of the LDL receptor family existing on the surface of rat podocytes, and have been proved to be related to endocytosis and cytokinesis of proteins (15). RAP can then bind to megalin to form a heterodimeric complex that assists in the folding of megalin in the endoplasmic reticulum and its transport to the cell surface, and it has been confirmed that RAP itself does not induce MN (16). Several epitopes on the heterodimeric complex appear to be involved in the formation of immune complexes (17). In animal models, active immunization with megalin alone or passive immunization with direct injection of relevant antibodies resulted in subepithelial deposition of immune complexes, but there was no C3 or C5b-9 deposition and no proteinuria (18). Antibodies against other parts of FxlA have been shown to inhibit complement regulatory proteins on podocytes, activating complement via the alternative pathway or the mannose-binding lectin pathway. Although some scholars have confirmed that megalin is expressed in human podocytes (19) and proximal tubular brush border (20), it can cause human anti–brush border antibody disease, which is characterized by renal failure, proximal tubule injury, and immune deposits in the tubular basement membrane containing LRP2 and IgG. However, it has not been found in glomerular immune complexes of MN patients, and no relevant antibodies have been found in the serum of the patient, which greatly limits the application of HN in human MN.

On the other hand, after the complement is exhausted by cobra venom factor, PHN model mice will not produce proteinuria, which confirms the key role of complement activation in the HN model. In PHN, complement is activated by classical and alternative pathways (21, 22), which was previously thought to be different from the pathogenesis of human MN (classical and lectin). However, recent research has confirmed the important role of the classical pathway in human MN (23). This has greatly improved the practical value of the PHN model.

These two models have their advantages and disadvantages: the AHN model has stable pathological changes, the glomerular function damage and proteinuria in the AHN rats are more serious than the PHN, but modeling time is long and changes greatly during this period. The PHN model is more suitable for studying the renal injury caused by proteinuria, because the kidney is the only damaged target organ in PHN model rats. The model has the advantages of stable pathological changes, rapid onset, short disease course and strong practicability. The pathological mechanism is similar to that of human beings, but it cannot meet the needs of long-term observation. Therefore, on the basis of a single intravenous injection of anti-FxlA antibody, low-dose antisera can be injected intraperitoneally multiple times to strengthen immunity. The 24-hour urinary protein of the model mice continued to increase, and the kidney injury was aggravated, which was convenient for dynamic observation of the kidney injury, pathogenesis of PHN disease and the treatment of MN drugs.

### 2.2 Anti-dipeptidyl peptidase IV rat model

Dipeptidase IV (DDP IV, gp 108) is a glycoprotein present in glomerular capillary loops, tubules and intestinal microvilli, and NATORI et al. (24) proved that it is one of the main antigens of FxlA. Rabbit anti-DPPIV was injected into rats, and rabbit IgG was deposited in glomerular capillary loops for 4-8 hours. Rats will develop proteinuria within 8 hours, reach a peak after 2 days (>200 mg/24 h), and then rapidly decline. Renal pathology shows glomerular IgG deposits but no C3 deposits, which is obviously different from human MN. Compared with HN, this model has a shorter duration of proteinuria and IgG deposition (most disappear within 5 days) and is not as practical as the HN model.

## 3 Mouse model

### 3.1 The cationic bovine serum albumin model

The charge barrier is an important part of the glomerular filtration barrier, which plays a vital role in the penetration of macromolecules in blood through the glomerular filtration barrier (25). In 1982, BORDER et al. (26) successfully induced MN by intravenous injection of C-BSA into New Zealand white rabbits and proved that antigen charge was the key factor for the formation of subepithelial immune complex. Some scholars have successfully induced MN in animals such as dogs (27), cats (28) and rats (29–31) by using this method. In 2004, CHEN et al. (32) successfully constructed the mouse C-BSA model by injecting C-BSA every other day intravenously in combination with Freund’s complete adjuvant into mice for one week and re-immunizing them two weeks later with C-BSA. Four weeks later, the experimental mice were sacrificed. Hyperlipidemia, hypoproteinemia and severe proteinuria occurred in mice after high dose injection of C-BSA, and the strong particle fluorescence of IgG and C3 was observed by immunofluorescence. The electron microscope showed the deposition of electron dense substance on the subepithelial surface and the disappearance of the podocyte foot process, and the level of IgG1 in serum was significantly higher than that of IgG2a. After that, the optimal dosage for inducing related MN model in different strains of mice — ICR was 7 mg/kg, BALB/c was 13 mg/kg, whereas it was not inducible in C57BL/6 mice. It also proves that a genetic background with a predisposition for Th2 cells may determine the successful induction and progression of MN. Since then, some scholars have repeatedly immunized BLAB/c mice with C-BSA (C-BSA, 13 mg/kg, tiw) for six weeks, and successfully established the C-BSA mouse model (33). In 2011, DEBIEC et al. (25) detected high levels of IgG1 and IgG4 subclasses of anti-bovine serum albumin antibodies and C-BSA in circulating form in the sera of some children with MN, and observed the co-localization of C-BSA and IgG in glomerular *in situ* immune complexes, which confirmed the pathogenic potential of C-BSA in human MN and could be used as an exogenous food antigen for children with MN.

### 3.2 Anti-α3 NC1 mouse model

The non-collagen 1 domain of human α3 (IV) collagen has been proven to be the main autoantigen against human anti-basement membrane glomerulonephritis (34, 35), and its expression in mouse podocytes has also been confirmed (36). In 2012, ZHANG J-J et al. (37) successfully constructed a related MN model by synthesizing the α3 chain non-collagenous region (rh-α3 (IV) NC1 domain) of human type IV collagen fibers and emulsifying them in an equal volume of Freund’s complete adjuvant and subcutaneously injecting them into DBA/1, FcγR −/−, and FcγRIII −/− mice, followed 3 weeks later by a booster immunization with Freund’s incomplete adjuvant mixed with α3 NC1, and the model mice showed massive proteinuria and nephrotic syndrome, subepithelial IgG and C3 deposition, glomerular basement membrane thickening, and diffuse disappearance of podocyte foot processes. Subsequently, it was found that twice immunizing DBA/1 mice with this method led to MN models, and four immunizations led to focal necrotic or crescentic glomerulonephritis based on MN, which was characterized by crescents formation, extensive tubulointerstitial injury and a large number of macrophage infiltration (38). In DBA/1 mice, compared with human α3 NC1, murine α3 NC1 was less effective in inducing autoimmunity, less crescent formation and tubulointerstitial injury, and would prolong the course of the disease for 1-2 weeks (39, 40).

The induction of nephropathy in this model is faster and more reliable than HN. However, the sensitivity of different mouse strains to this approach is obviously different, especially in C57BL/6 mice, which can only induce moderate proteinuria without nephrotic syndrome, which is also an aspect that needs to be solved urgently.

### 3.3 Anti-podocyte antibodies induced MN model

The PHN model in rats shows that antibodies against specific antigens on podocytes lead to the deposition of the subepithelial immune complex and the breakthrough of podocyte foot. Given this, MEYER et al. (41) established a model of MN induced by an anti-podocyte antibody. The team injected mice pre-immunized with Freund’s adjuvant 5 days before the experiment and intravenously injected polyclonal rabbit antibodies against the murine podocyte cell line. 7 days later, the mice developed proteinuria, which peaked on day 10. Immunoelectron microscopy showed that rabbit IgG and C3 were deposited linearly along the glomerular basement membrane and adjacent foot processes, but subepithelial deposition was not observed. Due to the limited number of rabbit anti-podocyte antibodies, the team selected different mouse podocyte cell lines as antigens (42, 43) and produced sheep anti-podocyte antibodies using the same method. Interestingly, goat antibodies were significantly improved compared with rabbit antibodies, which showed that podocyte foot process disappeared and significant subepithelial immune complex deposition was produced (44). C3 deletion/deficiency mice can also produce the above phenomenon, and the occurrence time and degree of proteinuria are parallel to normal mice, indicating that complement activation is not a necessary condition for the formation of proteinuria in this model, and there is a complement-independent mechanism. However, due to a large amount of ascites, mice are likely to develop acute renal failure secondary to nephrotic syndrome, so the mice need to be killed within 14 days after injection of antibody.

### 3.4 Anti-aminopeptidase A model

Anti-mouse aminopeptidase A antibody (mAb) is a hydrolase existing in the mice kidneys and participates in the degradation of angiotensin. The researchers (45) produced an anti-mouse aminopeptidase A antibody, also known as ASD-4, by fusing mouse myeloma cells with rat spleen cells immunized with the brush border membrane of the mouse kidney. ASD-4 belongs to subclass IgG1, and diffuse distribution of ASD-4 on S1, S2 segments of proximal tubular brush border and glomerular epithelial cell membrane can be observed by indirect immunofluorescence and immunoelectron microscopy in normal mice. After injecting ASD-4 into normal mice, mice showed dose-dependent proteinuria, and the antibodies were observed to bind evenly to the glomerular capillary wall and became granular one day later. Under the electron microscope, podocyte foot process fusion and electronic dense deposits were observed, but there was no GBM thickening and complement system activation (46), and proteinuria persisted for at least 16 days. Subsequent studies (47) showed that albuminuria in the anti-APA model was related to the structural changes of CD2-related protein (CD2AP) and nephrin, which played an important role in maintaining the structure of the slit diaphragm and podocyte function.

Similar to the models of anti-DDPIV and anti-podocyte antibodies, this model induced glomerular IgG deposition and proteinuria by passive antibody administration, but complement activation was rare, and the above antigens were not present in human PMN, so they were significantly different from human PMN.

### 3.5 Thrombospondin type-1 domain-containing 7A-associated MN model

In 2014, TOMAS et al. (48) identified thrombospondin type-1 domain-containing 7A (THSD7A) as a novel target antigen for human MN. THSD7A is a type 1 transmembrane protein located outside the podocyte membrane. Studies have demonstrated that this antigen is expressed in mouse podocytes (49), and this 250 kDa glycoprotein has been found to contain multiple regions containing epitopes (50). In China, it is associated with approximately 16% of PLA_2_R1-negative PMN patients (51). In 2016, TOMAS et al. (52) reported a patient with end-stage kidney disease with THSD7A-associated MN who rapidly relapsed after receiving a kidney transplant and later observed positive THSD7A staining in the transplanted kidney and anti-THSD7A antibodies were detected in the patient’s serum both before and after transplantation, which indicated that the same type of MN recurred. Therefore, by extracting anti-THSD7A antibodies from the patient’s serum and injecting it intravenously into mice, it was observed that human antibodies combined with mouse podocyte THSD7A antigen formed *in situ* immune complexes and deposited linearly along the glomerular capillary wall, and mice showed significant proteinuria around 3 days after injection, which continued to increase after 14 days, and significant proteinuria persisted until 70 days, which indicated that a mouse model closer to human MN was successfully constructed. The following year (53), the team co-immunized rabbits with a combination of murine THSD7A (mTHSD7A) and human THSD7A (hTHSD7A) by cDNA, and produced rabbit anti-THSD7A antibodies. Subsequently, mice receiving rabbit anti-THSD7A antibody purified from rabbit serum injected intraperitoneally into BALB/c mice developed severe nephrotic syndrome, including severe proteinuria, edema, and hyperlipidemia. Immunofluorescence showed that after 14 days of antibody injection, the granular antigen-antibody complexes deposited under the subepithelium in the glomerular filtration barrier of mice, which showed typical characteristics of MN in humans. Interestingly, activation similar to human IgG4 subclasses and complement was not detected in this model, but antibodies activated the lectin pathway in mice, which led to oxidative stress, destruction of nephrin, and rearrangement of the podocyte actin cytoskeleton (52–54).

Compared with previous MN models, this model has made remarkable progress —a mouse model with the same target antigen as human MN is constructed for the first time, and its pathological mechanism is closer to that of human PMN, which is of great significance for studying more real human PMN. Unfortunately, at present, this model has not been successfully replicated in commonly used experimental animals such as C57BL/6 mice, DBA/J1 mice and SD rats. In this regard, the researchers believe that rabbit anti-THSD7A antibody can activate Th2 subgroup in BALB/c mice and then produce significant MN-related manifestations, while it mainly activates Th1 subgroup in C57BL/6 mice (55, 56). In short, this greatly limits the practicability of this model and fails to be applied on a large scale.

### 3.6 M-type phospholipase A_2_ receptor 1 -related MN model

In 2009, BECK et al. (57) found that M-type phospholipase A2 receptor 1(PLA_2_R1) is the main target of human membranous nephropathy (MN), and this finding has greatly promoted basic and clinical research. Primary membranous nephropathy is nowadays considered a limiting autoimmune disease of the kidney, with antibodies against PLA_2_R1 (aPLA_2_R1ab) found in 70-80% of patients of various ethnic groups. However, many related issues, such as the development of its autoimmune response, the role of IgG subclasses and epitopes, and the pathway of podocyte injury, need to be explained. The development of PLA_2_R1-related membranous nephropathy is most likely influenced by genetic susceptibility, loss of tolerance, and changes in antigen expression, but environmental factors such as air pollution, heavy metal poisoning, smoking and infection also play an important role (58). However, PLA_2_R1 is not expressed in glomeruli of wild-type rats and mice (59, 60), so conventional methods such as antibody injection could not produce the MN model. Interestingly, MOELLER et al. (61) reported a transgenic mouse strain that only expressed Cre recombinase in podocytes, which is regulated by a 2.5 kb fragment of the human NPHS2 promoter. This fragment was shown to drive β-galactosidase (β-gal) expression only in podocytes of transgenic mice. Histological analysis of the kidneys showed that the expression of β-gal was confined to podocytes. In view of this, MEYER et al. (62) adopted the transgenic knock-in method in 2020 to inject the gene of Rosa26/CAG/Stop/mPLA_2_R1 into the target vector (63) (mice) embryonic stem cells into blastocysts. Breeding with NPHS2-Cre (or PodoCre) mice allowed podocyte-specific expression of mouse PLA_2_R1 (mPLA_2_R1), followed by breeding of mice that were homozygous for both the mPLA_2_R1 allele and the PodoCre allele, and this transgenic mouse expressed mPLA_2_R1 in podocytes. Expression of mPLA_2_R1 did not lead to the morphological disorder of the foot process. High-resolution confocal microscope and electron microscope showed that all mice had normal foot process, glomerular basement membrane and endothelial cells. In addition, the optical microscope examination showed that the overall morphology of glomeruli and tubulointerstitial remained unchanged. The researchers then synthesized rabbit anti-mPLA_2_R1 and injected it into the abdominal cavity of transgenic mice. These mice rapidly developed proteinuria, with an albumin/creatinine ratio of 100 g/g. The serum cholesterol levels increased significantly, while serum urea nitrogen levels remained normal. There was a dose-dependent relationship between proteinuria and injected, and proteinuria could last for about 21 days, during which the albumin/creatinine ratio decreased slowly. Histological examination showed granular deposition of IgG, widened foot process, and partial co-localization of complement C3 and IgG. To this point, the team successfully constructed a mouse model of mPLA_2_R1-associated MN. Subsequently, the team tried to inject patient-derived anti-PLA_2_R1 IgG into some mPLA_2_R1 positive mice, but the results were negative. No antigen-antibody binding was observed and MN could not be induced.

Similar to the THSD7A model, this model induced a disease model with similar manifestations to human PMN by passive antibody administration, accompanied by deposition of IgG and complement C3 in the glomerular subepithelium and a large number of proteinuria, which introduced the experimental model into the era of common target antigens of MN in humans and mice. It has been confirmed that human PMN antigens such as Semaphorin 3B (Sema3B) and HtrA Serine Peptidase 1 (HTRA1) are expressed in mouse podocytes (59, 64). Whether we can use similar methods to combine immune adjuvants to enhance the susceptibility of mice and then induce MN in other antigen categories remains to be further studied. Unfortunately, the mechanism of passive antibody administration inducing MN cannot explain the mechanism of autoantibody production in human PMN.

### 3.7 The hPLA_2_R1 associated model

In order to address the question of different sources of antigen, MEYER et al. (65) successfully cultured mice expressing the human PLA_2_R1 antigen (hPLA_2_R1). After 3 days of birth, strong expression of hPLA_2_R1 was observed in the membrane and cytoplasm of mouse glomerular podocytes. The electron microscope confirmed that a complete glomerular filtration barrier was visible 7 days after birth, and no obvious glomerular or tubulointerstitial changes were observed by light microscope, and the glomerular structure was intact. Transgenic mice showed no significant proteinuria after 1 week of birth. Spontaneous anti-hPLA_2_R1 antibodies were detected in the peripheral blood of positive mice after 2 weeks. Until 4 weeks old, IgG (mIgG) deposition and complement activation were observed in the glomeruli of positive mice, and typical proteinuria, hyperlipidemia, and hypoproteinemia appeared, which became more and more serious with the increase of time. After 6 weeks, mIgG was deposited in granular form on the glomerular basement. The electron microscope showed that podocyte foot process effacement, subepithelial electron dense deposits and glomerular basement membrane thickening.

In this model, mice spontaneously produced anti-hPLA_2_R1 antibodies, which recognized cysteine-rich domains and C-type lectin domains 1, 7 and 8, which were epitopes recognized by human anti-PLA_2_R1 antibodies (66–68). More importantly, model mice can spontaneously produce diseases similar to those of human PMN, without relying on exogenous antibodies and more truly simulate the state of epitope mutation or exposure in human PMN, which is of great significance for studying the initial pathogenesis of PMN and is an ideal model to clarify the pathogenesis and antibody pathogenicity of human PMN. It remains to be investigated whether a non-PLA_2_R1 antigen-related MN model can be reproduced using similar methods. However, within 3-4 weeks after the appearance of anti-PLA_2_R1 antibody, severe nephrotic syndrome and an accelerated course of end-stage kidney disease appeared in the model mice, which cannot meet the needs of long-term observation.

### 3.8 The chPLA_2_R1 associated model

In view of the fact that the expression of hPLA_2_R1 will cause a large amount of proteinuria in transgenic mice in a short time, researchers have to kill the mice at 6 weeks old. However, transgenic mice expressing mPLA_2_R1 are well tolerated. Therefore, researchers attempt to construct a chimeric mouse model which can produce proteinuria and other manifestations of MN to meet the needs of long-term observation. Therefore, they hypothesized that a chimeric PLA_2_R1(chPLA_2_R1) which is rich in three highly immunogenic N-terminal human domains (CysR、Fnll and CTLD1) (66, 69, 70) and the seven more C-terminal murine domains CTLD2-8 would be tolerated by mice. Rosa26/CAG/Stop/chPLA_2_R1 gene was knocked into the R1 embryonic stem cells of the target vector by a method similar to the previous model (62), and then transgenic mice with specific expression of chPLA_2_R1 were produced (71). The phenotype of transgenic mice is normal and there is no obvious abnormal behavior. In the first six months after birth, there was no difference in body weight, urinary albumin-to-creatinine ratios, serum albumin and blood urea nitrogen levels between chPLA_2_R1-positive and -negative mice. The expression of chPLA_2_R1 was not found in other organs except the kidney. Histological examination showed that chPLA_2_R1 was strongly expressed in the cytoplasm of mouse podocytes and partially co-localized with nephrin near the podocyte foot process. No glomerular, renal tubular and interstitial damage was found in PAS staining, and the glomerular filtration barrier was intact under the electron microscope. No circulating antibody against hPLA_2_R1, chPLA_2_R1 or the murine part of chPLA_2_R1 mCTLD2-8 in the serum of chPLA_2_R1-positive mice and no glomerular murine IgG (mIgG) deposits. Next, the research team actively immunized these mice with the extracellular part of hPLA_2_R1 to verify whether transgenic mice can produce MN. ChPLA_2_R1 positive male mice aged 12-14 weeks were injected with a mixture (60ul) of 1 ug of recombinant extracellular hPLA_2_R1 diluted in a 0.9% NaCl and the mild adjuvant TiterMax Gold with a ratio of 1:1. 21 days later, the mice were boosted with 1 ug recombinant extracellular hPLA_2_R1 diluted in 0.9% NaCl and TiterMax Gold at a 4:1 ratio. The mice were weighed every week, and the levels of urine protein and serum albumin were detected. The levels of urea nitrogen, triglyceride and cholesterol were continuously monitored daily. The results showed that the ratio of urinary albumin to creatinine in the experimental group increased compared with the baseline level after 3 weeks of immunization with hPLA_2_R1. At the 5th week, anti-hPLA_2_R1 antibodies could be detected in the serum of transgenic mice, and their constructs with hPLA_2_R1, including hCysR-FnII, hCTLD1-2 and hCTLD7-8, could be detected. Albuminuria substantially increased around 6 weeks after immunization with hPLA_2_R1. Some animals developed severe proteinuria and required a sacrifice of the animals 12 weeks ago. Morphological examination showed the typical manifestations of MN, including the disappearance of podocyte foot process, the deposition of electron dense substance in the subepithelial layer, the thickening of GBM and the formation of spike process. After 12 weeks, the granular deposition of mIgG under GBM, which was strongly co-localized with chPLA_2_R1, was observed. At the same time, the researchers found that mIgG1 is the richest subtype of mIgG in the glomerular immune complex of transgenic mice. Previous studies have shown that mIgG1 could not bind C1q and activate the complement system through the classical pathway, which is similar to human IgG4 (hIgG4) (23, 72), showing that this model has common ground with human PMN in the aspect of complement activation.

In a word, the antigen specificity, immune-mediated character, and dominance of non-C1q-binding mIgG1 in combination with the typical clinical model and morphology in regard to mIgG and complement deposition can be considered as the major advantages of this new experimental model of PLA_2_R1-related MN. The model mice have a long course of disease, which can meet the needs of long-term observation, making it the most suitable animal model of membranous nephropathy at present.

## 4 Non-rodent model of MN

Compared with rodent models, non-rodents are relatively seldom used because of the high cost of feeding, long modeling and breeding cycles. Several non-rodent models of MN have been developed, including:

### 4.1 The cationic bovine serum albumin model

C-BSA models in rabbits, dogs and cats (as discussed before) are not commonly used now.

### 4.2 Neutral endopeptidase models

NEP and DDPIV are both antigens present in brush borders and podocytes and participate in the formation of subepithelial deposits in animal models. Studies have shown that these two antigens are expressed in human podocytes (73, 74). In 2002, DEBIEC et al. (75) reported a case reported a case in which the anti-NEP antibodies were transferred from the pregnant woman to the fetus, resulting in severe MN in the baby. Then, the researchers injected IgG from the infant’s parents intravenously into rabbits and induced a disease similar to MN, which was characterized by obvious proteinuria and IgG deposition along the capillary wall.

### 4.3 Angiotensin-converting enzyme antibody-induced MN model in minipigs

In the investigation of the humoral consequence of pig-primate xenotransplantation, researchers observed that in minipigs injected with heterologous antibody of angiotensin converting enzyme, heterologous IgG was linearly or granularly bound to the basement membrane, glomerular subepithelial immune complex deposition, and renal tubular lesions, and the model pigs showed mildly increased proteinuria (76).

### 4.4 PLA_2_R1-related minipig models

**Figure 2:**
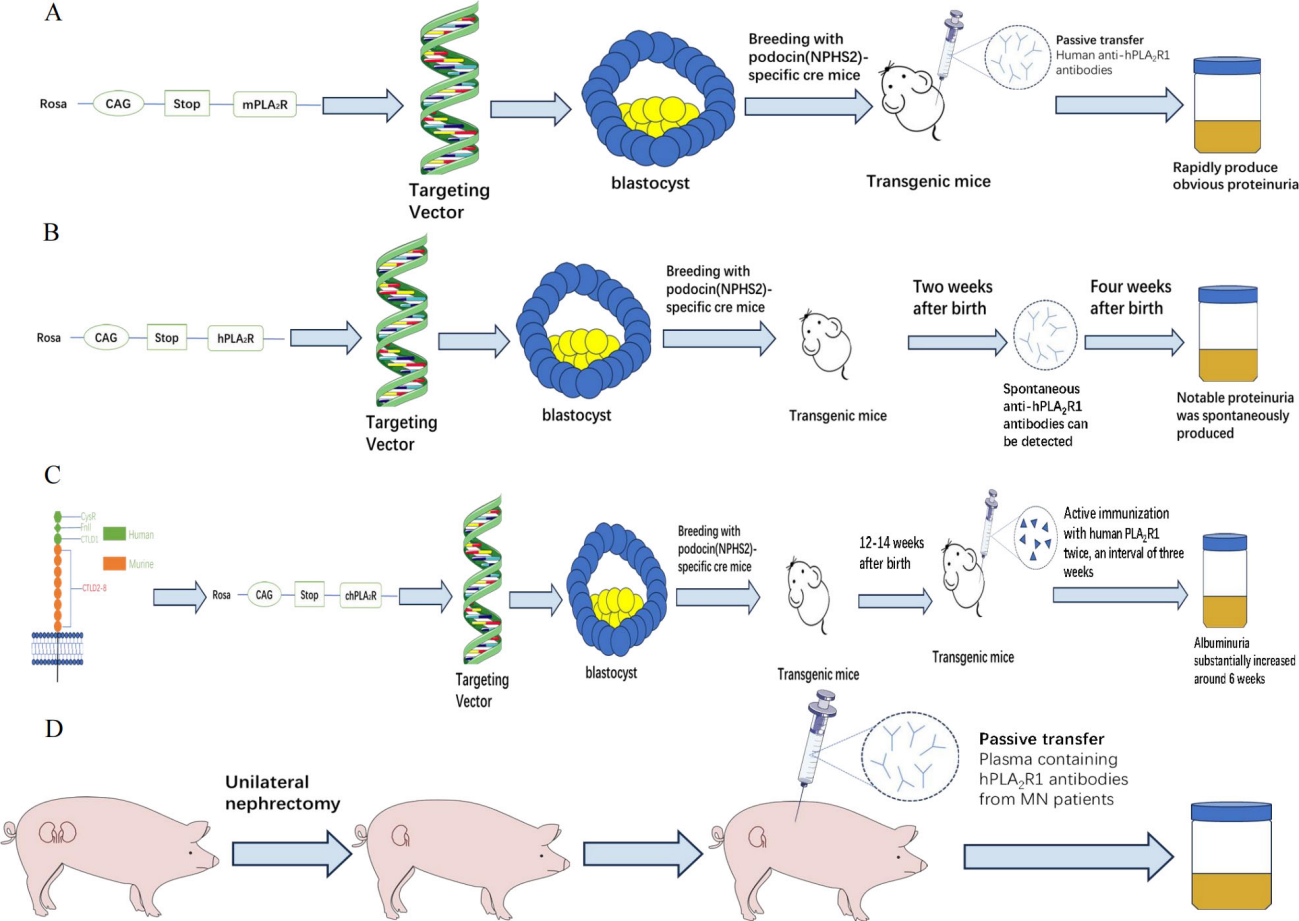
Establishment of four kinds of animal models of PLA2R1. **(A)** The mPLA2R1-related model; **(B)** The hPLA2R1 associated model; **(C)** The chPLA2R1 associated model; **(D)** The PLA2R1-related minipig models; PLA2R1, phospholipase A2 receptor 1; m, mouse; h, human; ch, chimeric.

Recently, REINHARD et al. (77) found that PLA_2_R1 antigen expressed in podocytes of minipig can bind to anti-hPLA_2_R1 from patients. So the researchers first performed unilateral nephrectomy on experimental minipigs to increase antigen exposure, then injected plasma containing hPLA_2_R1 antibodies from MN patients into the experimental group, while plasma from healthy people was injected into the control group, and then hPLA_2_R1 antibodies were only detected in the glomerulus of the experimental group. Within 7 days after injection, the urinary side of the glomerular basement membrane of minipig A showed fragmented and distinct granular positivity for human IgG (hIgG) and porcine C3. Electron microscopy showed subepithelial electron dense deposits associated with podocyte foot process effacement in a pattern reflecting stage I MN in patient biopsies. The experimental animals showed low-level proteinuria. This further confirmed the pathogenicity of the human PLA_2_R1 antibody in MN. Unfortunately, after injecting the patient’s plasma, the minipigs failed to produce the corresponding clinical symptoms and histological manifestations in a short time, which may be related to the lack of antibodies and the influence of other components in the plasma on MN in experimental animals cannot be ruled out. At the same time, due to the lack of antibodies, the experiment only studied single animals and the sample size was obviously insufficient. (**Figure 2**; **Table 2**).

**Table 2:** Similarities and differences of PLA2R1 related MN models.

	The mPLA2R1-related model	The hPLA2R1 associated model	The chPLA2R1 associated model	The PLA2R1-related minipig models
Animal	PodoCre mice expressing mPLA2R1	PodoCre mice expressing hPLA2R1	PodoCre mice expressing chPLA2R1	minipig
Antibody source	Exogenous antibody	Endogenous antibody	Endogenous antibody	Exogenous antibody
Antibody	Rabbit anti-mPLA2R1 antibodies	Mice anti-hPLA2R1 antibodies	Mice anti-hPLA2R1 antibodies	Human anti-PLA2R1 antibodies
Histological manifestation	Granular deposition of IgG, widened foot process, and partial co-localization of complement C3 and IgG	Granular deposition of mIgG in the subepithelial layer, disappearance of podocyte foot process, thickening of glomerular basement membrane.	Disappearance of podocyte foot process, deposition of electron dense substance in the subepithelial layer, thickening of GBM and formation of spike process.	Fragmented and distinct granular positivity for human IgG (hIgG) and porcine C3, subepithelial electron dense deposits, podocyte foot process effacement
IgG subclasses	Unknown	mIgG1>mIgG2>mIgG3	mIgG1>mIgG2>mIgG3	hIgG4
Proteinuria	Rapidly produce obvious proteinuria; last for about 7days.	Notable proteinuria was spontaneously produced after 4 weeks of birth.	Appeared after 3 weeks after immunization, substantially increased around 6 weeks, a chance of severe proteinuria 12 weeks ago	Low-level proteinuria within 7 days
Complement	C3, C5b-9	C3, C1q, C4d, CFB, CFH, C5b-9 et al.	C1q, C4d, CFB, C3, C5 et al.	C1q, CFB, C3, C5-C9 et al.

## 5 Summary

The ideal MN model is helpful for understanding the pathogenesis, treatment and prognosis of human MN. At present, the animal models of MN have gone through three stages: deposition of exogenous antigens - passive administration of antibodies - and spontaneous production of MN, of which passive administration of antibodies includes two different aspects: targeting animal-derived antigens and human-animal common target antigens. The above models can mimic human membranous nephropathy to some extent, but whether they can completely replicate the characteristics of human membranous nephropathy remains to be further studied.

To sum up, the recently constructed animal models of PLA_2_R1 and THSD7A are undoubtedly the best choice for studying the clinical manifestations or pathological mechanisms of membranous nephropathy, especially the recently constructed hPLA_2_R1 associated model. However, the above animal models are difficult to construct and obtain, and are only studied on a small scale at present, so they cannot be popularized for the time being. Therefore, it is still a good choice for researchers to adopt previous animal models. Among them, the HN rat model (especially the PHN rat model) has stable lesions and rapid onset, and its lesions are similar to human membranous nephropathy, which has been widely studied, so it has high recognition in the world. At the same time, in view of its similar complement activation pathway to human PMN, PHN model has obvious advantages in studying complement pathogenesis and complement-related drugs in patients with PMN (78, 79). Unfortunately, the HN model was successfully established only in rats, while the C-BSA animal model has been successfully established in rats, mice, rabbits and other species, and the preparation of the model is simpler than the HN model. For the preparation of large-scale animal model, considering the cost and feasibility, this model may have more advantages, and several recent studies have confirmed its important role in the drug treatment of membranous nephropathy, it has become an important extension and supplement of HN model (80, 81). In addition, there are still some problems with the preparation of animal models: the success rate of various modeling methods is not ideal, the modeling methods in different animals and even different strains of mice are poor in repeatability, the comparability between different animal models is poor, and the establishment process is complicated. Therefore, the existing animal models of membranous nephropathy is not entirely satisfactory. In order to further clarify the pathogenesis of membranous nephropathy and establish more effective treatment strategies, the animal models of membranous nephropathy need further improvement and exploration.
